# Endoscopic CO_2_ Laser Excision of Internal Pyolaryngocele: A Rare Airway Emergency Case Report With Review of the Literature

**DOI:** 10.1002/ccr3.71893

**Published:** 2026-01-27

**Authors:** Luigi Falchetta, Mario Carucci, Matteo Calvanese, Alfonso Scarpa, Giovanni Salzano, Francesco Antonio Salzano

**Affiliations:** ^1^ Department of Medicine, Surgery and Dentistry, “Scuola Medica Salernitana” University of Salerno Salerno Italy; ^2^ A.O.U San Giovanni di Dio e Ruggi d'Aragona U.O.C. Clinica Otorinolaringoiatrica Salerno Italy; ^3^ Maxillofacial Surgery Unit Federico II University of Naples Naples Italy

**Keywords:** airway emergency, case report, laryngocele, laryngopyocele, pyolaryngocele

## Abstract

A pyolaryngocele is a rare, potentially life‐threatening complication of laryngocele, resulting from secondary infection and obstruction of the saccular neck. Clinical severity ranges from mild dysphonia to acute airway compromise. We report a 51‐year‐old man with a sore throat, dysphagia, and dyspnea. Flexible laryngoscopy and contrast‐enhanced CT revealed an internal pyolaryngocele. After emergency tracheostomy and intravenous antibiotics, the patient underwent CO_2_ laser microlaryngoscopic excision and marsupialization. Postoperative recovery was uneventful, with complete healing and no recurrence at follow‐up. This case underscores the importance of early diagnosis and highlights CO_2_ laser surgery as an effective, minimally invasive option. Internal pyolaryngocele is a rare airway emergency that requires prompt management. CO_2_ laser excision appears to be a safe and effective first‐line approach in selected internal cases.

## Key Clinical Message

Early recognition of internal pyolaryngocele is crucial to prevent life‐threatening airway compromise. In internal pyolaryngocele, CO_2_ laser microlaryngoscopic excision offers a safe, effective, and minimally invasive treatment, ensuring rapid recovery, low morbidity, and excellent functional outcomes while minimizing recurrence risk. Prompt diagnosis and tailored surgical management are essential.

## Graphical abstract

Graphical abstract illustrating a large internal pyolaryngocele causing airway narrowing, diagnosed on contrast‐enhanced CT scan.

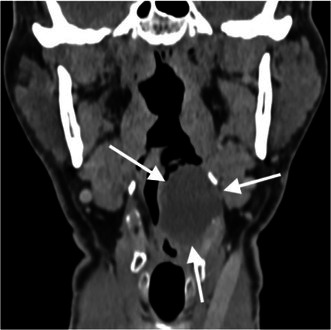

## 1 Introduction

**FIGURE 1:**
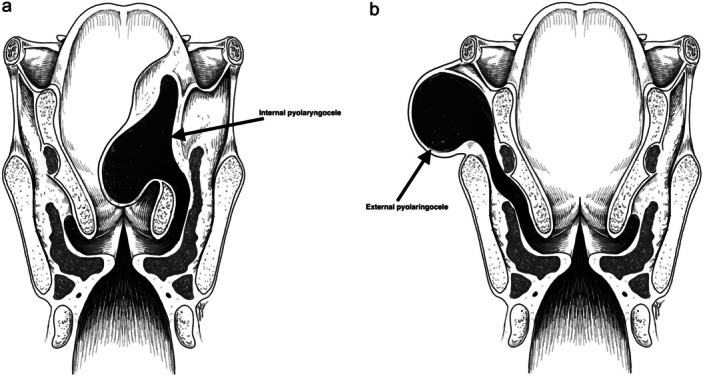
(a and b) Anatomical classification of laryngoceles [1].

A laryngocele is a rare benign air‐ or fluid‐filled dilation of the laryngeal saccule, commonly resulting from chronically elevated intralaryngeal pressure or congenital susceptibility. Classified as internal, external, or mixed based on extension through the thyrohyoid membrane, laryngoceles may rarely become secondarily infected and form a pyolaryngocele (Figure 1).

This is clinically significant due to the risk of airway obstruction and sepsis, with an estimated incidence of 1 per 2.5 million per year [2, 3]. It can be present at any age, but is most common in the sixth decade of life [1, 2, 4, 5, 6, 7, 8, 9].

We report a rare case of internal pyolaryngocele in a middle‐aged adult, managed successfully with CO_2_ laser microlaryngoscopic excision.

## 2 Case History

**FIGURE 2:**
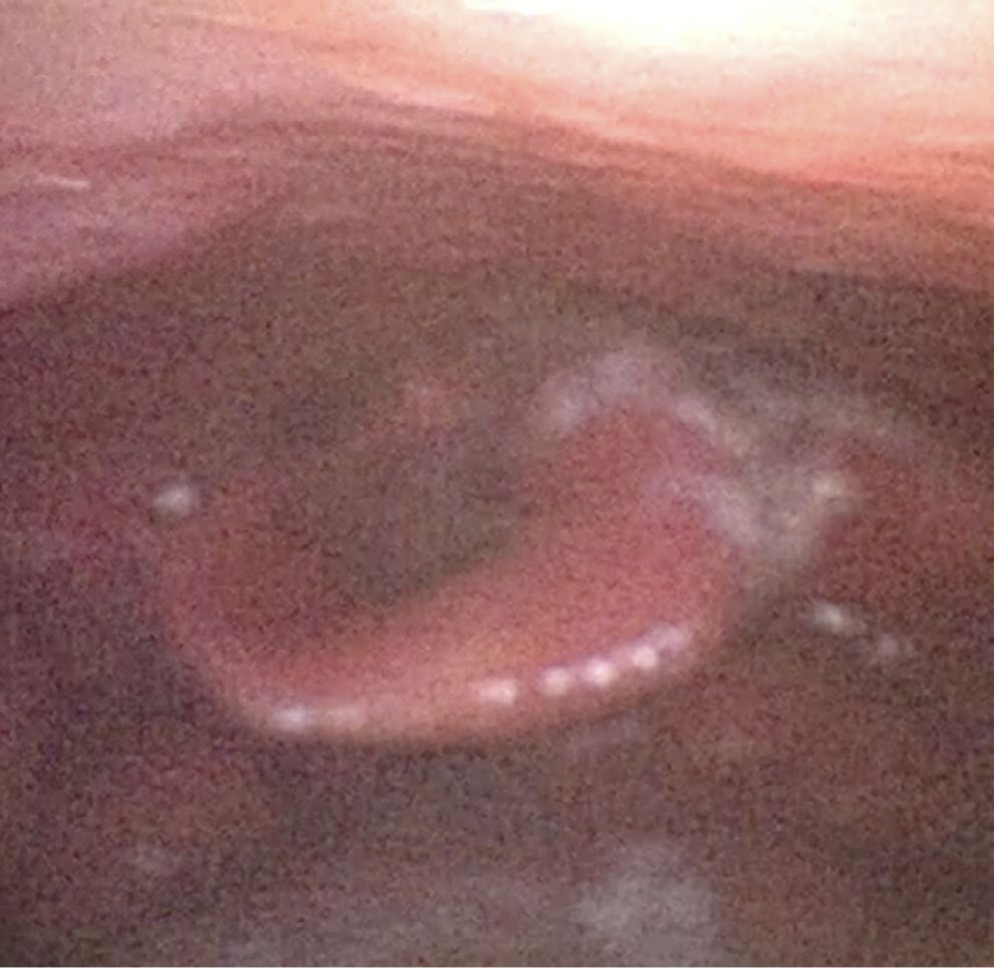
Laryngoscopy showing medial bulging of the right aryepiglottic fold.

A 51‐year‐old male gardener, an active smoker (20 cigarettes/day), with only a prior epiglottic history of abscess drainage, presented with a 20‐day history of sore throat, progressive dysphagia, and inspiratory dyspnea. Laryngoscopy showed medial bulging of the right aryepiglottic fold, partially obliterating the piriform sinus (Figure 2).

## 3 Differential Diagnosis, Investigations and Treatment

**FIGURE 3:**
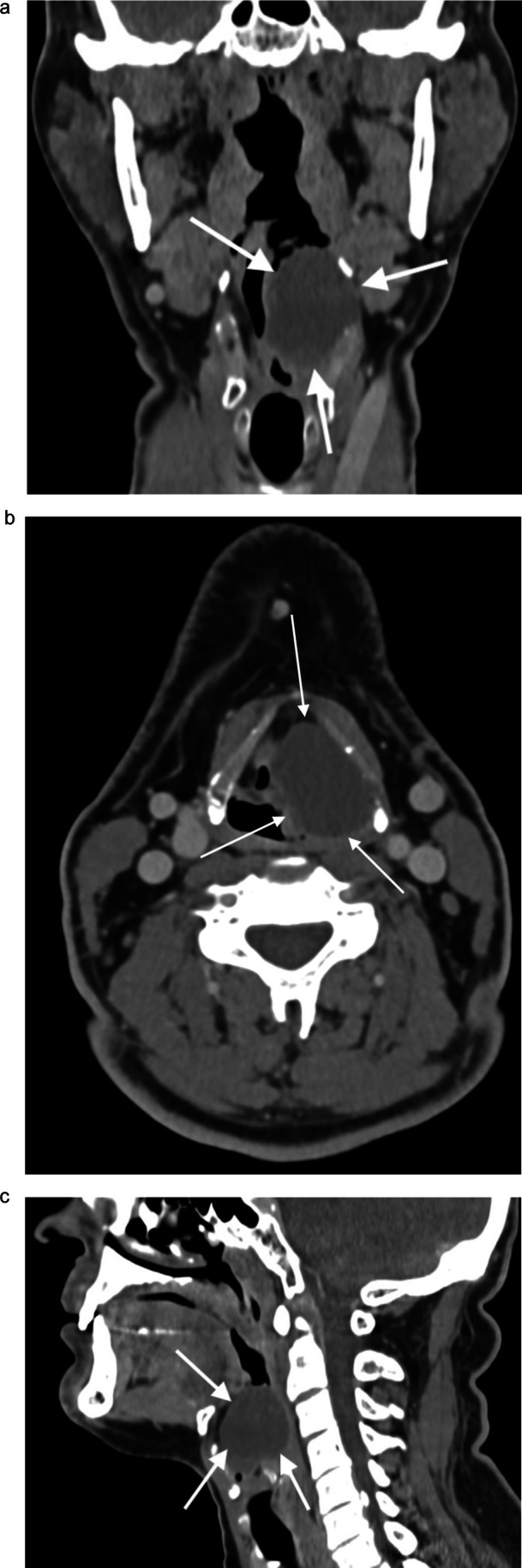
Coronal (a), axial (b), and sagittal (c) computer tomography scan revealed a hypodense cystic lesion consistent with an internal pyolaryngocele.

Contrast‐enhanced CT (Figure 3a–c) demonstrated a 3.2 × 2.5 cm hypodense cystic lesion extending caudally to 4 cm, displacing the airway to the right and abutting the thyroid cartilage, consistent with an internal pyolaryngocele [4].

The patient received intravenous ceftriaxone (2 g/day) and metronidazole (1500 mg/day). Due to acute airway compromise and failed intubation, an emergency tracheostomy was performed. Microlaryngoscopy revealed purulent supraglottic collection, which was incised and drained. After recurrence at 48 h, CO_2_ laser microlaryngoscopic excision and marsupialization were performed.

## 4 Conclusion and Results

**FIGURE 4:**
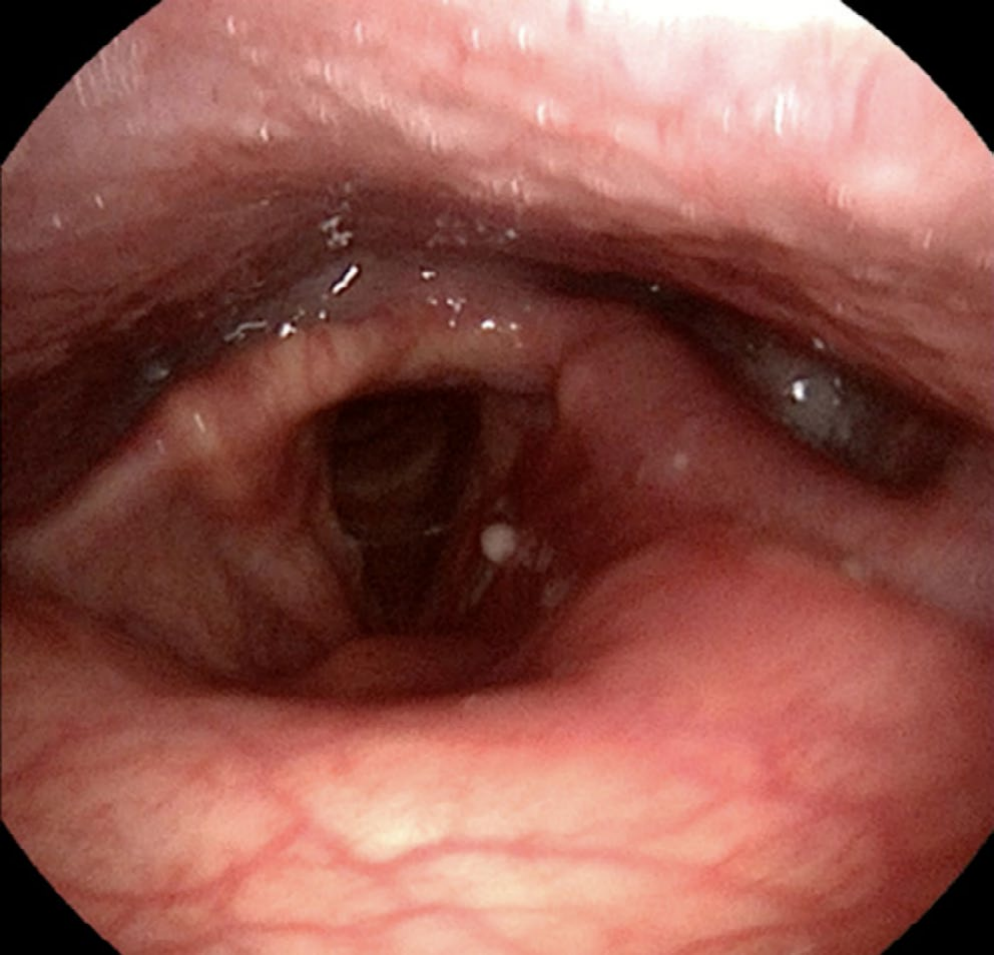
Follow‐up laryngoscopy showing complete healing with no recurrence.

Postoperatively, recovery was uneventful. Tracheostomy was removed on day 2; discharge followed on day 4. Endoscopic follow‐up confirmed complete healing with no recurrence (Figure 4). Histopathology showed pseudostratified columnar epithelium over fibromuscular connective tissue, without malignancy, consistent with laryngocele [5].

After discharge on day 4, the patient underwent scheduled postoperative evaluations at 7 days, 1, 3, 6, and 9 months. Flexible laryngoscopy at each follow‐up confirmed complete resolution of the infection, adequate mucosal healing, and absence of recurrence. In addition, the patient reported no further symptoms throughout the follow‐up period.

## 5 Discussion and Literature Review

Laryngoceles arise from the pathological distension of the laryngeal saccule, typically lined with mucus‐secreting pseudostratified ciliated epithelium. Obstruction by inflammation or infection may result in pus accumulation, forming a pyolaryngocele—a rare but potentially life‐threatening entity due to supraglottic airway compromise [1, 2, 3]. It's estimated that 8%–10% of laryngoceles may become infected at some point during their natural history [3, 10]. Although most laryngoceles remain asymptomatic, infection can lead to rapid enlargement and onset of symptoms such as hoarseness, sore throat, dysphagia, cervical swelling, and stridor [11]. Pyolaryngocele should be considered in the differential diagnosis of any rapidly progressive supraglottic swelling, especially in patients with known risk factors such as smoking or prior upper airway infections [6]. However, cases without identifiable risk factors have also been described [12]. Maweni et al. [13] highlighted that misdiagnosis as a parapharyngeal abscess can delay treatment. Cassano et al. [9] highlighted a possible association between laryngopyocele and underlying laryngeal carcinoma, stressing the need for malignancy exclusion.

Diagnosis relies on flexible fiberoptic laryngoscopy for direct visualization and contrast‐enhanced CT for anatomical mapping and surgical planning [6, 7, 14, 15]. Nazaroglu et al. [15] described typical CT features—such as wall thickening, rim enhancement, and air‐fluid level—that help distinguish pyolaryngocele from other deep neck masses. In selected emergency settings, point‐of‐care ultrasound has also been reported as a rapid diagnostic tool when CT is unavailable [16] and ultrasound may also guide percutaneous drainage in selected patients [17].

CT imaging is essential to distinguish between internal, external, and mixed forms [14, 15, 18] and to assess the degree of airway obstruction. Initial treatment includes broad‐spectrum antibiotics and airway management, often requiring tracheostomy when intubation fails [1, 19]. Fröhlich et al. [1], and Sabat et al. [7] both described episodes of acute airway obstruction and stridor, underscoring the emergency nature of this condition [1, 7, 20, 21, 22] and the importance of early intervention. Vasileiadis et al. [20] also described severe airway compromise requiring emergency intervention. Delayed diagnosis can be fatal, as demonstrated by Beautyman et al. [23] in one of the earliest lethal reports of untreated laryngopyocele.

Surgical drainage is necessary for purulent collections. Definitive management depends on lesion characteristics. For internal lesions, endoscopic CO_2_ laser excision offers high precision, minimal morbidity, and excellent functional preservation. Singh et al. [3] emphasized the utility of minimally invasive techniques in their review, while Fraser et al. [8] described the successful use of a microdebrider to manage an infected laryngocele without the need for tracheostomy. For extensive or recurrent pyolaryngoceles, complete excision via an external approach remains the gold standard, as confirmed by Marharay et al. [24]. Mahdoufi et al. [4, 5] and Touihmi et al. [2] also reported favorable outcomes with external approaches for mixed or large cases.

Although conservative management has been proposed in selected asymptomatic cases [25], surgery remains the standard.

Heuveling and Mahieu [26] further expanded the role of endoscopic management by demonstrating that even large combined laryngoceles can be excised transorally using the CO_2_‐laser “inversion technique,” in which the external component is gradually pulled inward and turned inside‐out through the thyrohyoid membrane.

Our case adds to the limited reports demonstrating that CO_2_ laser marsupialization is a safe and effective treatment for internal pyolaryngocele, with rapid recovery and no recurrence. However, this approach requires specialized equipment and expertise and may be less effective in large or mixed‐type lesions [6, 8]. Although open surgery, such as trans‐thyrohyoid or V‐shaped thyrotomy, remains an option in selected cases, the trend toward endoscopic management continues to grow due to reduced morbidity and favorable outcomes.

## 6 Conclusion

Internal pyolaryngocele is a rare ENT emergency requiring prompt multidisciplinary management. This case highlights successful CO_2_ laser microlaryngoscopic excision following airway stabilization and antibiotics, supporting its role as a first‐line, minimally invasive approach in selected patients.

## Author Contributions

**Luigi Falchetta:** conceptualization, investigation, writing – original draft. **Mario Carucci:** validation. **Matteo Calvanese:** resources. **Alfonso Scarpa:** supervision, writing – review and editing. **Giovanni Salzano:** supervision. **Francesco Antonio Salzano:** project administration.

## Funding

The authors have nothing to report.

## Consent

Written informed consent was obtained from the patient for publication of this case report and accompanying images.

## Conflicts of Interest

The authors declare no conflicts of interest.

## Data Availability

The data that support the findings of this study are available from the corresponding author upon reasonable request.
